# Astroglial Connexin43 as a Potential Target for a Mood Stabiliser

**DOI:** 10.3390/ijms22010339

**Published:** 2020-12-30

**Authors:** Motohiro Okada, Tomoka Oka, Misaki Nakamoto, Kouji Fukuyama, Takashi Shiroyama

**Affiliations:** Department of Neuropsychiatry, Division of Neuroscience, Graduate School of Medicine, Mie University, Tsu 514-8507, Japan; otomoka008@gmail.com (T.O.); m-nakamoto@clin.medic.mie-u.ac.jp (M.N.); k-fukuyama@clin.medic.mie-u.ac.jp (K.F.); takashi@clin.medic.mie-u.ac.jp (T.S.)

**Keywords:** depression, bipolar disorder, mood stabiliser, astrocytes, connexin, tripartite synaptic transmission

## Abstract

Mood disorders remain a major public health concern worldwide. Monoaminergic hypotheses of pathophysiology of bipolar and major depressive disorders have led to the development of monoamine transporter-inhibiting antidepressants for the treatment of major depression and have contributed to the expanded indications of atypical antipsychotics for the treatment of bipolar disorders. In spite of psychopharmacological progress, current pharmacotherapy according to the monoaminergic hypothesis alone is insufficient to improve or prevent mood disorders. Recent approval of esketamine for treatment of treatment-resistant depression has attracted attention in psychopharmacology as a glutamatergic hypothesis of the pathophysiology of mood disorders. On the other hand, in the last decade, accumulated findings regarding the pathomechanisms of mood disorders emphasised that functional abnormalities of tripartite synaptic transmission play important roles in the pathophysiology of mood disorders. At first glance, the enhancement of astroglial connexin seems to contribute to antidepressant and mood-stabilising effects, but in reality, antidepressive and mood-stabilising actions are mediated by more complicated interactions associated with the astroglial gap junction and hemichannel. Indeed, several depressive mood-inducing stress stimulations suppress connexin43 expression and astroglial gap junction function, but enhance astroglial hemichannel activity. On the other hand, monoamine transporter-inhibiting antidepressants suppress astroglial hemichannel activity and enhance astroglial gap junction function, whereas several non-antidepressant mood stabilisers activate astroglial hemichannel activity. Based on preclinical findings, in this review, we summarise the effects of antidepressants, mood-stabilising antipsychotics, and anticonvulsants on astroglial connexin, and then, to establish a novel strategy for treatment of mood disorders, we reveal the current progress in psychopharmacology, changing the question from “what has been revealed?” to “what should be clarified?”.

## 1. Introduction

Recent neuropharmacological and psychopharmacological studies emphasised the importance of the modulation of tripartite synaptic transmission for the treatment of various neuropsychiatric disorders [1,2,3,4,5,6,7,8,9,10,11,12,13]. Tripartite synaptic transmission has traditionally referred to glutamatergic transmission between neurones and astrocytes, whereas, recently, the conception of tripartite synaptic transmission has been extended to other transmission systems, such as monoaminergic tripartite synaptic transmissions [14]. Taken together with the monoaminergic hypothesis, which is one of the most established pathophysiological hypotheses of mood disorders [15], the extended conception of monoaminergic tripartite synaptic transmission suggests that astrocytes are probably involved in the pathomechanisms of mood disorders.

Indeed, several clinical studies have reported the possibilities associated with astroglial dysfunctions in individuals with major depression [16,17,18,19,20,21,22,23]. Postmortem studies demonstrated the abnormalities associated with astrocytes in corticolimbic regions. The reduction of the glial population in the dorsolateral prefrontal cortex, orbitofrontal cortex [16], subgenual cortex [17], anterior cingulate cortex [18], and amygdala [19] of individuals with major depression was reported in numerous studies; however, an increase in glial size was also observed in major depression [20,21] but not in schizophrenia [24,25]. Based on these postmortem findings, reduced glial density being compensated for by glial cell enlargement has traditionally been considered a specific response of mood disorders. This hypothesis was supported by preclinical studies that showed that both chronic unpredictable stress and prolonged social defeat reduced the expression of glial fibrillary acidic proteins [26,27,28]. Additionally, astrocytes contribute to energy metabolism, including glucose transport and glycogenolysis in the central nervous system [29,30]. Astroglial glycogenolysis plays essential roles in K^+^ uptake [31]. Indeed, clinical findings using positron emission tomography and functional magnetic resonance imaging indicated the dysfunction of glucose metabolism as well as impaired function in brain regions involved in emotional processing and cognitive functions (the prefrontal cortex, amygdala, and hippocampus) in individuals with major depression, suggesting astrocyte hypoactivity in cognition-promoting regions [22,23]. Contrary to in major depression, several studies also reported glial abnormality but reduced glial population and size in the brains of patients with bipolar disorder [17,32,33,34,35]. These discrepancies of astroglial abnormalities between major depression and bipolar disorder suggest the possibility that the pathomechanisms of these mood disorders are not identical. In spite of these efforts, the detailed mechanisms of glial abnormality associated with mood disorders remain to be clarified.

Neuron‒glial crosstalk consists not only of extracellular tripartite synaptic transmission, but also of intracellular pan-neuroglial networks via the gap junction [1,36,37,38]. Both hemichannel and gap junction provide the numerous astroglial functions, including both physiological and pathological conditions [1]. The hemichannel and gap junction are constructed by connexin, which is a family of 21 protein isoforms [1,39,40]. Six connexin units assemble to form homomeric or heteromeric connexons [1]. Two connexons in two neighbouring cells form a gap junction with an aqueous pore and charged surface walls [1], whereas a single connexon contributes to a chemical connection between the intra- and extra-cellular spaces as a hemichannel [1]. The transmembrane pores of the connexon are permeable to ions, metabolites, second messengers, mRNA, and purine signalling mediators up to 1.5 kDa [41]. In steady state, cultured astrocytes are characterised by a high level of gap junctional communication and low hemichannel activity [42].

Gap junctions provide the intracellular communications associated not only with rapid exchange of cations, transmitters, and second messengers, which are fundamental molecules for electrophysiological excitability and its propagation, but also with prolonged physiological processes, cellular development, and homeostasis [1,39,40]. In particular, the astroglial gap junction contributes to the cytoplasm-to-cytoplasm communication of biochemical or ionic mobilisation between a cell and adjacent cells, leading to the regulation of ionic and several other types of homeostasis via the regulation of intracellular Ca^2+^ mobilisation and K^+^ buffering [1,43,44]. Therefore, the physiological function of connexin provides the maintenance of various astroglial homeostasis system in the central nervous system [1,39,40]. Contrary to physiological conditions, pathological hyperactivated conditions generate persistent hemichannel opening, which leads to the disruption of several homeostasis systems [1,3,45,46]. During the resting stage, the hemichannel exhibits low opening probability, whereas depolarisation, ischemia, specific cation mobilisation, and connexin phosphorylation activate the hemichannel, resulting in the persistent astroglial nonexocytotic release of excitatory L-glutamate, D-serine, adenosine triphosphate, kynurenine metabolites, and eicosanoids [1,2,3,9,12,47]. Therefore, astrocytes participate in tripartite synaptic transmission not only via exocytosis but also by nonexocytotic gliotransmitter release through the hemichannel [1,3,6,7,8,9,12].

Connexin43 (Cx43) is the most widely and predominant expressed connexin subtype in the central nervous system, including the astrocyte [1]. Accumulating evidence suggests that functional abnormalities of Cx43 play key roles in the pathophysiology of mood disorders. A recent behavioural study reported that genetic inactivation of Cx43 enhanced the antidepressant action of acute fluoxetine administration [48]. However, the functional abnormality of Cx43 associated with mood disorders is more complicated than expected. It seems that the pathophysiology of mood disorders cannot be fully elucidated without a systematic examination of Cx43 kinetics, the functions of the Cx43 gap junction, and the hemichannel. Based on the above clinical and preclinical findings, in this review, we introduce clinical findings regarding the abnormality of Cx43 in mood disorders, and discuss the potential of Cx43 as a target of mood-stabilising medication in the pathophysiology of mood disorders associated with Cx43.

## 2. Abnormalities of Cx43 in Depression

Various postmortem studies demonstrated that Cx43 expression in the locus coeruleus, frontal cortex, mediodorsal thalamic nucleus, and caudate nucleus of patients with major depression was reduced compared to healthy individuals (Table 1) [49,50,51,52]. These clinical findings suggest that the dominant regions of Cx43 expression abnormality are not only mood/emotional but also cognitive regulation regions [1,7,8,10,53,54,55,56,57,58]. Therefore, these postmortem studies indicated that decreased Cx43 expression in the cortex, locus coeruleus, and thalamus plays important roles in the pathomechanisms of depressive mood or depressive emotional perception. Unlike in major depression, there are no postmortem studies exploring Cx43 expression’s association with bipolar disorder.

According to the stress hypothesis of depression, there are a number of depressive-like experimental animal models, including social stress, chronic mild and unpredictable stress, learned helplessness, early-life stress models, and exogenous corticosterone [53]. Preclinical studies demonstrated that three depression models—chronic unpredictable stress [59,60,61,62], restraint stress [63], and exogenous corticosterone [64,65]—affected either the expression or function of Cx43 (see detail in Section 3).

Acute restraint stress did not affect astroglial Cx43 expression but enhanced hemichannel opening probability in the mouse hippocampus [63]. The enhancement of hemichannel opening probability induced by acute resistant stress was augmented by chronic restraint stress [63]. The *N*-methyl-d-aspartate (NMDA)/glutamate receptor antagonist 3-(2-carboxypiperazin-4-yl)-propyl-1-phosphonic acid inhibited stress-induced hippocampal glutamate release [63]. These observations suggest that stress-induced hyperactivation of excitatory transmission through Cx43 hemichannel probably participates in the pathomechanisms of stress-induced mood disorders according to the NMDA/glutamate hypothesis of depression [53]. Indeed, a reduced gap junction and activated hemichannel are observed during exposure to severe stress, and hyperactivation of the Cx43 hemichannel generates the disassembled gap junction [66].

Chronic unpredictable stress and chronic administration of exogenous corticosterone are two experimental animal models of treatment-resistant depression [53]. Chronic unpredictable stress decreases Cx43 expression and suppresses gap junction permeability in the rat prefrontal cortex [59]. Chronic unpredictable stress decreased gap junction density, resulting in the inhibition of astroglial communication in the prelimbic cortex [59], and increased endogenous corticosterone [67]. These observations suggest that the increase in endogenous corticosterone induced by chronic unpredictable stress probably contributes to a decrease in Cx43 expression. Indeed, chronic administration of exogenous corticosterone also reduces Cx43 expression in rat cultured astrocytes [65] via the enhancement of degradation and suppression of Cx43 synthesis [68]. Another study reported that the contradictive effects of chronic corticosterone administration increase phosphorylated Cx43 expression without affecting the expression of total Cx43 in the hippocampus [64,68]. In particular, corticosterone phosphorylates Cx43 at Ser368 [68,69], which inhibits the function of Cx43 containing a gap junction [70] via the augmentation of gap junction internalisation and degradation [71].

Therefore, the previous findings demonstrated by postmortem and experimental animal model studies indicate that the suppression of expression and function of astroglial Cx43 contributes to the pathomechanisms of depressive mood.

## 3. Cx43 and Behaviour

A sucrose preference test demonstrated that local administration of the nonselective gap junction/hemichannel inhibitor carbenoxolone (CBX) into the prefrontal cortex caused significant decreases in consumed sucrose, indicating CBX-induced anhedonia [59]. A novelty-suppressed feeding test also demonstrated that CBX prolonged latency to feed, indicating CBX-induced anxiety-like behaviour [59]. The other inhibitors, Cx43-selective mimetic peptide inhibitors Gap27 and Gap26, displayed similar effects to CBX [59]. These findings strongly indicate that the inhibition of Cx43 containing a gap junction/hemichannel in the frontal cortex plays important roles in the pathophysiology of depression and anxiety.

Unlike in the prefrontal cortex, constitutive deficiency of Cx43 in hippocampal astrocytes did not affect behaviour in the sucrose preference test, whereas it did decrease immobility time in the tail suspension test, indicating antidepressant-like behaviour [64]. Both elevated plus maze and open field tests also showed that local administration of CBX to the bilateral ventral hippocampus (but not dorsal hippocampus) reduced anxiety-like behaviour [73]. Interestingly, a unilateral local administration of CBX into the ventral hippocampus plus a contralateral local administration to the medial prefrontal cortex showed similar antidepressant-like effects [73]. Cx43-knockout mice also displayed a decrease in latency to feed in a novelty-suppressed feeding test without indication of abnormalities in elevated plus maze and open field tests [64] (Table 2).

Given the findings of postmortem and experimental animal model studies, reduced Cx43 kinetics in regions other than the hippocampus appears to be associated with pathomechanisms of depression; however, the effects of hippocampal Cx43 on mood disturbance require detailed consideration to clarify the mechanisms, since the results are inconsistent. Acute/chronic restraint stress activates corticosterone response (release) [74] and hippocampal hemichannel activity [63], whereas exogenous corticosterone suppresses hippocampal gap junction activity [68]. The discrepancy in the effects between inhibition of hippocampal and frontal connexins suggests that Cx43 kinetics alone cannot explain the pathomechanisms of mood disturbance. In other words, in the hippocampus, activation of hemichannel activity probably plays important roles in the pathomechanisms of depressive mood compared with Cx43 kinetics (reduced Cx43 expression) and gap junction function.

## 4. Effects of Monoamine Transporter-Inhibiting Antidepressants on Cx43

In vivo study without any stress stimulation and chronic administrations of fluoxetine [59,76] and duloxetine [59] consistently increased the mRNA and proteins of Cx43 in the frontal cortex. Chronic administrations of fluoxetine and duloxetine also compensated for the decreased expression of mRNA and proteins of Cx43 induced by chronic unpredictable stress in the frontal cortex [59]. Interestingly, chronic administration of fluoxetine (18 mg/kg/day for 28 days) suppressed corticosterone-induced phosphorylated Cx43 expression in the hippocampus and depressive-like behaviours [64]. Therefore, both fluoxetine and duloxetine at least partially activate the expression and function of hippocampal Cx43 (Table 3).

In vitro cultured astrocyte studies without any stress stimulation, and subacute administrations (24–48 h) of fluoxetine, paroxetine, duloxetine, venlafaxine, reboxetine [75], milnacipran, and cocaine [77], did not affect Cx43 expression; however, the gap junction activity of cortical astrocytes was enhanced by fluoxetine, duloxetine [59], and paroxetine [75] apart from in one report [75]. A noteworthy finding is that all antidepressants, fluoxetine, paroxetine, reboxetine, duloxetine, and venlafaxine, inhibited cortical astroglial hemichannel activity induced by lipopolysaccharides [75] (Table 3).

It has been established that, under physiological conditions, cortical cultured astrocytes and acute slices are characterised by high levels of gap junctional communication and low hemichannel permeability [78,79]. Regarding the Cx43 kinetics, the antidepressant action of monoamine transporter-inhibiting antidepressants possibly consists of activation of gap junction function (including secondary increased quantities of gap junction function due to increased Cx43 expression) and/or inhibition of hemichannel activity induced by pathological stimulation such as stress or proinflammatory reaction.

## 5. Effects of Antipsychotics and Ketamine on Cx43

The noncompetitive NMDA/glutamate receptor inhibitor ketamine/esketamine is effective for treatment-resistant major depression, suicidal ideation, and anhedonia [53]. Numerous clinical trials have demonstrated that ketamine, a noncompetitive NMDA/glutamate receptor antagonist, could evoke a rapid onset of antidepressive action (within several hours) [53,81,82,83]. The major mechanism of antidepressive action of ketamine/esketamine is considered to be inhibition of the NMDA/glutamate receptor, resulting in GABAergic disinhibition [53]; however, a preclinical study demonstrated that acute administration of ketamine (for 30 min) concentration-dependently inhibited the permeability of the astroglial gap junction and hemichannel [84]. In particular, the inhibitory effects of ketamine on astroglial hemichannel activity (threshold concentration: 20–50 µM) were dominant compared to on the gap junction (threshold concentration: 300 µM) [84]. Ketamine had anaesthetic and antidepressive effects in humans and rats at concentrations of 10 and 20 µM, respectively [53,85,86]. Therefore, acute administration of a therapeutic-relevant concentration of ketamine inhibited hemichannel activity but not the gap junction. Considering the effects of monoamine transporter-inhibiting antidepressants and ketamine in astroglial transmission associated with connexin, the clinical efficacy of ketamine in treatment-resistant depression leads to the interesting hypothesis that the inhibition of astroglial hemichannel activities contributes to antidepressive action (Table 4).

Thus far, the effects of mood-stabilising antipsychotics on astroglial Cx43 in the central nervous system of patients with schizophrenia remain to be clarified. With regard to the effects of antipsychotics on astroglial Cx43, only haloperidol, clozapine, and olanzapine have been assessed [3,8,76,77,84]. An initial in vivo study could not identify consistent effects of antipsychotic class on frontal Cx43 expression, since chronic administration of haloperidol, clozapine and olanzapine decreased, increased, and did not affect frontal Cx43 expression, respectively [76]. In vitro studies using cortical cultured astrocytes also demonstrated that subacute haloperidol and chronic clozapine administration did not affect or increased astroglial Cx43 expression, respectively [3,77]. The stimulatory effect of clozapine on astroglial Cx43 expression in the plasma membrane was predominant compared to that in a cytosol fraction [3]. The functional analysis study also demonstrated that acute (for 60 min) and chronic (for seven days) administrations of therapeutic-relevant concentration of clozapine did not affect astroglial basal l-glutamate release, but enhanced astroglial l-glutamate release through the activated Cx43 hemichannel [3,8]. Therefore, the stimulatory effects of clozapine on astroglial glutamatergic transmission [1,8,13] are probably mediated by the enhancement of functionally activated Cx43 hemichannel in the plasma membrane [3,8]. Therefore, the effect of clozapine on astroglial Cx43 expression was similar to that of the monoamine transporter-inhibiting antidepressants, but the effect of clozapine on astroglial hemichannel permeability was opposite to that of antidepressants, including ketamine and monoamine transporter-inhibiting antidepressants. Recent meta-analysis studies and systematic reviews have emphasised the mood-stabilising effects of clozapine [87,88,89,90]. Clozapine can improve both psychotic and affective symptoms, whether in an acute or maintenance phase [90]. Furthermore, another meta-analysis and systematic reviews reported that the efficacy of clozapine was similar to that of other antipsychotics in manic episodes, but superior to other antipsychotics for treatment-resistant bipolar disorder [88,89]. Considering the clinical features of clozapine, ketamine, and monoamine transporter-inhibiting antidepressants, an enhancement of function of cortical astroglial hemichannel probably contributes to the mechanisms of antimanic action (Table 4).

There is a paucity of reports on whether the effects on astroglial Cx43 expression or function are involved in the mechanism of efficacy of olanzapine in bipolar disorder; thus, more detailed studies are needed in the future. In particular, the combination of olanzapine with lithium (Li) or valproate is considered to be the first choice for the treatment of acute manic phases of bipolar disorder [91,92]. A recent systematic review reported that olanzapine is considered a maintenance treatment for bipolar disorder [93]. Based on the evaluation of the effectiveness of a combination therapy of olanzapine with Li or valproate, the correlation between the effect of olanzapine alone and the combination of olanzapine with Li or valproate on Cx43 is important for understanding the pathophysiology of bipolar disorders.

## 6. Effects of Anticonvulsants on Cx43

Traditionally, epidemiological studies reported that the suicide rate in patients with epilepsy is 5-fold higher than in the general population, whereas in patients with temporal lobe epilepsy/complex partial seizures, it is 25-fold higher [94,95]. A certain psychiatric comorbidity may provoke suicidality in patients with epilepsy [94,95,96]. Furthermore, the depressive mood and cognitive impairment often linked with epilepsy or adverse anticonvulsant reactions seem to be major risks for suicidality in patients with epilepsy [94,95,96]. Some anticonvulsants enhance serotonergic transmission, i.e., carbamazepine, lamotrigine, topiramate, valproate, and zonisamide [97,98,99,100,101,102,103,104,105]. The stimulatory effects of these anticonvulsants on serotonergic transmission can explain, at least partially, their mood-stabilising action [106]; however, it is well known that depressive adverse reactions of these anticonvulsants are not rare [94,95]. Therefore, the pathomechanisms of depressive comorbidity in patients with epilepsy and depressive adverse anticonvulsant reactions require another working hypothesis that includes novel target molecules other than serotonergic transmission.

It has also been established that functional abnormalities of astrocytes play important roles in the development of epileptogenesis, since several recent clinical and preclinical studies indicated the hyperactivation of Cx43 in an epileptic focus region of refractory temporal lobe epilepsy, type IIB focal cortical dysplasia, kindling rats, and a genetic animal model of idiopathic epilepsy [4,5,46,107,108,109]. However, contrary to expectations, initial studies using cultured astrocytes did not detect significant effects of subacute administrations (24‒48 h) of carbamazepine, valproate, gabapentin, phenytoin, or diazepam on Cx43 expression in total lysate [77,110]. Contrary to subacute administration, the chronic administration (seven days) of therapeutic-relevant concentration of anticonvulsants on astroglial Cx43 seemed complicated. Chronic administration of therapeutic-relevant concentrations of zonisamide suppressed Cx43 expression in astroglial plasma membrane and hemichannel opening probability [2]; however, chronic administration of therapeutic-relevant concentrations of lacosamide suppressed Cx43 hemichannel function without affecting astroglial Cx43 expression in the plasma membrane [2]. Chronic administration of therapeutic-relevant concentrations of carbamazepine did not affect astroglial Cx43 expression in the plasma membrane or Cx43 hemichannel activity, but supratherapeutic concentrations of carbamazepine inhibited both Cx43 expression in the plasma membrane and hemichannel permeability [2]. These three first-line anticonvulsants used for the treatment of focal epilepsy are categorised in the voltage-dependent sodium channel (VDSC) inhibitor class [3,111]. The dissimilar effects of these three anticonvulsants on the function and expression of astroglial Cx43 possibly explain the pathophysiology of carbamazepine-resistant focal epilepsy, along with mood and cognitive adverse reactions. Indeed, a number of clinical studies have reported that lacosamide and zonisamide can inhibit CBZ-resistant focal epilepsy syndrome [112,113,114,115] (Table 5).

Lacosamide leads to a lower risk of cognitive disturbance than carbamazepine and zonisamide [116,117]. Additionally, some clinical studies reported the beneficial effects of lacosamide in patients with moderate or severe depression [118]. Contrary to lacosamide, zonisamide has been reported to exhibit antimanic efficacy but a high risk of depression [96,119]. Together with the clinical features of lacosamide and zonisamide, inhibition of astroglial hemichannel function contributes to antidepressant-like effects, which is supported by the effects of monoamine transporter-inhibiting antidepressants and ketamine; however, despite the inhibition of the hemichannel, the inhibition of Cx43 expression or gap junction function induced by reduced Cx43 expression possibly leads to depressive mood (Table 5).

Chronic valproate administration also affects cortical astroglial Cx43 expression, but its effects appear to be distinctly different from those of the above VDSC-inhibiting anticonvulsants [3]. The VDSC-inhibiting anticonvulsants carbamazepine, lacosamide, and zonisamide inhibited the expression of Cx43 in the plasma membrane or Cx43-containing hemichannel activity in a concentration-dependent manner within the range from therapeutic-relevant to supratherapeutic concentrations, whereas valproate increased the expression of astroglial Cx43 expression in the total lysate without affecting that in the plasma membrane [2,3]. The transcription of Cx43 is regulated by epigenetic processes, including histone modifications, DNA methylation, and microRNA species [1,43,120]. Valproate inhibits class I and IIa isoforms, with histone deacetylase resulting in the increase in the various gene expressions [121,122]. Indeed, several histone deacetylase inhibitors, suberoylanilide hydroxamic acid, trichostatin A, and 4-phenylbutyrate, increased the expression of Cx43 mRNA and protein [123,124,125]. The mood-stabilising effect of valproate has been proven to be more effective in acute manic episodes and the maintenance of bipolar disorder rather than bipolar depression, in combination with several atypical antipsychotics [126,127,128]. Similar to monoamine transporter-inhibiting antidepressants, valproate possibly enhances the astroglial gap junction function through increased expression of Cx43 during soundness or depressive states. On the other hand, the increased intracellular Cx43 induced by valproate can augment the stimulatory effects of antipsychotics such as clozapine on trafficking Cx43 to the plasma membrane of hemichannel activity.

**Table 5:** Summary of the effects of first-line mood stabilisers and other anticonvulsants on the expression and function of Cx43.

Agent	Model (Region)	Treatment (Dose, Duration)	Effect (Hemichannel)	Reference
Carbamazepine	rat cortical astrocyte	in vitro (40–400 µM for 24 h)	no effect (protein)	[110]
	rat cortical astrocyte	in vitro (100 µM for 7 days)	no effect (protein) (no effect)	[2]
Lacosamide	rat cortical astrocyte	in vitro (30–100 µM for 7 days)	no effect (protein) (inhibition)	[2]
Zonisamide	rat cortical astrocyte	in vitro (30 µM for 7 days)	decrease (protein) (inhibition)	[2]
Valproate	Rat (frontal)	in vivo (300 mg/kg for 21 days)	no effect (protein)	[76]
	rat cortical astrocyte	in vitro (350–1400 µM for 24 h)	no effect (protein)	[110]
	rat cortical astrocyte	in vitro (1000–3000 µM for 7 days)	increase (protein) (activation)	[3]
Gabapentin	rat cortical astrocyte	in vitro (60–600 µM for 24 h)	no effect (protein)	[110]
Phenytoin	rat cortical astrocyte	in vitro (40–400 µM for 24 h)	no effect (protein)	[110]
Diazepam	rat cortical astrocyte	in vitro (25 µM for 48 h)	no effect (protein)	[77]

We searched MEDLINE using the keywords “(((connexin) OR (hemichannel) OR (gap junction)) AND ((anticonvulsant) OR (psychotropic drugs)))” for papers published by 1 November 2020. Relevant articles were obtained in full and assessed for inclusion independently by reviewers. Disagreements among reviewers were resolved via discussion to reach a consensus.

## 7. Candidate Pathophysiology of Mood Disorders associated with Cx43

**Figure 1:**
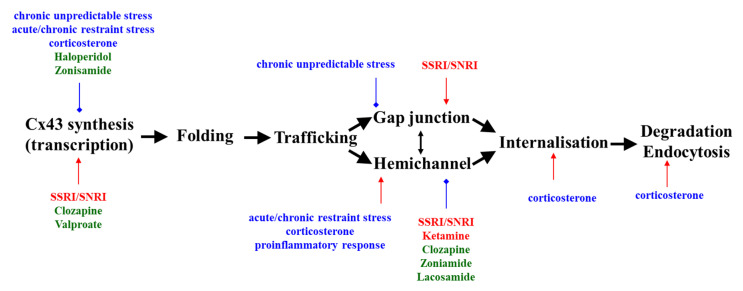
Summary of the effects of antidepressants (red), mood stabilisers (green), and depressive mood-inducing stress (blue) on astroglial Cx43. Red and blue arrows indicate stimulatory and inhibitory effects, respectively. Cx43: connexin43, SSRI: selective serotonin re-uptake inhibitor, SNRI: serotonin norepinephrine reuptake inhibitor.

A summary of the effects of depressive mood-inducing stresses, antidepressants, mood-stabilising anticonvulsants, and antipsychotics on astroglial Cx43 is shown in Figure 1.

### 7.1. Candidate Pathophysiology of Major Depression Associated with Cx43

Postmortem studies consistently reported a reduction in Cx43 expression in numerous brain regions of patients with depression (Table 1). The reduced astroglial Cx43 expression in the hippocampus and frontal cortex was also supported by several experimental depression rodent models, as well as chronic unpredictable stress, acute/chronic restraint stress, and exogenous corticosterone models (Table 2). Furthermore, in spite of reduced expression, these depressive mood-inducing stresses led to contradictive astroglial functional alternations between suppressed gap junction and enhanced hemichannel activities. Exogenous corticosterone exposure suppresses the density and permeability of the astroglial gap junction, but enhances hemichannel activities, with increased phosphorylated Cx43 [68,69]. Remarkably, phosphorylated Cx43 at Ser368 suppresses the gap junction containing Cx43 [70] via augmentation of gap junction internalisation and degradation, resulting in decreased astroglial Cx43 expression [71]. In other words, depressive mood-inducing stress enhances phosphorylated Cx43, leading to an attenuation of homeostatic gap junction function with stimulation of the toxic hemichannel function. Therefore, Cx43 is probably a terminal but fundamental target molecule in the hypothalamic–pituitary–adrenal hypothesis of the pathophysiology of depression.

Pharmacodynamic demonstrations of monoamine transporter-inhibiting antidepressants and ketamine support the dysfunction of Cx43, impaired gap junction, and activated hemichannel. Monoamine transporter-inhibiting antidepressants increase astroglial Cx43 expression and enhance gap junction function, but decrease phosphorylated Cx43 and reduce hemichannel activity [59,64,75,76]. Similar to monoamine transporter-inhibiting antidepressants, ketamine, which is a rapid-acting antidepressant that exhibits efficacy for the treatment of treatment-resistant depression, also inhibits astroglial hemichannel permeability (activity) without affecting the gap junction [84]. These results suggest that as a compensation for the activated hemichannel, the suppressed gap junction and decreased Cx43 expression induced by depression-inducing stress stimulation probably contribute to antidepressant-like action [59,64,75].

In studies of the pathophysiology of depression, the suppression of gap junction function and activation of hemichannel function in astrocytes were observed during depressive mood, whereas, conversely, monoamine transporter-inhibiting antidepressants and ketamine enhance astroglial gap junction function and inhibit hemichannel function. Therefore, the improvement of hypoactivity of astrocytes through the suppression of pathologically activated hemichannels is, at least partially, involved in the antidepressant-like action due to the improvement of dysfunction of homeostasis maintenance systems.

### 7.2. Candidate Pathophysiology of Other Mood Disorders Associated with Cx43

Compared to depression, our knowledge of the pathophysiology of bipolar disorder has not progressed due to a lack of postmortem studies regarding Cx43 kinetics and established experimental animal models of bipolar disorder. Studies of the pathophysiology of bipolar disorder should be dependent on the pharmacodynamic profiles of mood stabilisers; however, even the pharmacodynamic profiles of mood-stabilising antipsychotics and anticonvulsants have not provided sufficient findings to illuminate the pathophysiology of bipolar disorder.

Subacute administration of haloperidol did not affect Cx43 expression, whereas chronic haloperidol administration decreased [76,77]. Contrary to haloperidol, the mood-stabilising antipsychotic clozapine [88,89] chronically increased Cx43 expression [3,8,76]. Additionally, clozapine enhanced the function of activated astroglial hemichannel [3,8]. In steady state, cultured astrocytes are characterised by a high level of gap junctional communication and low hemichannel permeability [42]. Therefore, chronic exposure to therapeutic-relevant concentrations of clozapine probably enhances both astroglial gap junction and hemichannel. The stimulatory effects of clozapine on Cx43 expression are predominantly mediated by the post-transcriptional system, since the level of Cx43 in the plasma membrane was higher than that in the cytosol [3,8]. The stimulatory effects of clozapine on Cx43 expression in the astroglial plasma membrane are possibly modulated by the activation of signalling of protein kinase B (PKB) [1,46,129,130]. Contrary to clozapine, chronic administration of therapeutic-relevant concentrations of valproate weakly increases Cx43 in the cytosol but does not affect Cx43 in the plasma membrane through inhibition of histone deacetylase (activation transcription process of Cx43) [3]. Valproate monotherapy is effective for the acute phase of bipolar disorder; however, the efficacy of valproate is inferior to that of olanzapine [131]. Chronic administration of a combination of clozapine and valproate drastically increased Cx43 expression in the plasma membrane [3].

Although an in vivo study failed to detect effects of olanzapine on Cx43 expression [76], it is interesting to ponder the mechanism of the mood-stabilising effects of olanzapine associated with the interaction between PKB and Cx43. Olanzapine monotherapy and adjunctive olanzapine with valproate have established efficacy for bipolar disorder in the acute phase [93,132]. Meanwhile, the efficacy of adjunctive olanzapine and fluoxetine has been confirmed for acute phase and bipolar depression, respectively [93,132]. Single, acute administration of therapeutic-relevant doses of olanzapine and fluoxetine weakly activates PKB, whereas the administration of high doses of a combination of olanzapine and fluoxetine drastically enhances PKB activity [133]. Taken together with the effects of clozapine on Cx43 expression, the enhancement of PKB activity is a possible underlying mechanism of the mood-stabilising action of clozapine and olanzapine. The combination of PKB-activating agents, such as clozapine and olanzapine, with a reasonable adjunctive agent that synergistically promotes Cx43 function is expected to enhance the mood-stabilising effect. To clarify this hypothesis, we report on the interaction between valproate and several antipsychotics.

The increase in Cx43 expression induced by valproate seems to be disadvantage for the anticonvulsive action of valproate, whereas the therapeutic-relevant concentration of valproate weakly increases Cx43 in the cytosol but does not affect Cx43 in the plasma membrane through the transcription process [3]. Unlike valproate, both zonisamide and lacosamide suppress astroglial hemichannel activity [2]. Therapeutic-relevant concentrations of lacosamide, which are effective in patients with focal epilepsy comorbid with depression [118], suppress the astroglial function of the hemichannel without affecting astroglial Cx43 expression [2]. The clinical efficacy of lacosamide for depressed patients with focal epilepsy can be explained by the hemichannel-inhibiting hypothesis. Therapeutic concentrations of zonisamide, which exhibits antimanic efficacy but a depressive adverse reaction [96,119], suppress Cx43 expression in the astroglial plasma membrane with inhibiting the function of hemichannel activity [3]. The different effects of zonisamide and lacosamide on astroglial Cx43 and mood suggest that the inhibition of the hemichannel and gap junction contribute to antidepressive and antimanic effects, respectively. To clarify this hypothesis, we report the effects of other anticonvulsants, topiramate, lamotrigine, and oxcarbazepine, on astroglial Cx43 kinetics and function.

### 7.3. Potential of Cx43 as a Target for Mood Stabilisers

In this review, antidepressants suppress astroglial hemichannel activity and probably enhance gap junction activity, whereas mood-stabilising antipsychotics enhance both astroglial hemichannel and gap junction. These seem to be part of the pathophysiology of mood disorders: the inhibition and activation of astroglial hemichannel activities contribute to antidepressive and antimanic action, respectively. However, considering the effects of mood-stabilising anticonvulsants on astroglial Cx43, the effects of Cx43 on mood are possibly more complicated than we expected. Cx43 synthesis-enhancing agents, monoamine transporter-inhibiting antidepressants, and clozapine exhibit antidepressant-like action, whereas the Cx43 synthesis-inhibiting agents haloperidol and zonisamide exert antimanic-like action or depressive mood induction. Furthermore, lacosamide, which inhibits astroglial hemichannel activity without affecting Cx43 synthesis, had a mild but antidepressant-like effect rather than depressive mood induction [116,117,118]. Taken together with the clinical findings of postmortem and imaging studies [16,17,18,19,20,21,22,23], we note that the relative enhancement of astroglial gap junction function due to increased Cx43 synthesis possibly compensates for the hypoactive neuronal or tripartite synaptic transmission in the mood/emotional/cognitive promoting regions. Indeed, a nonselective connexin inhibitor, CBX, and Cx43-selective mimetic peptide inhibitors Gap27 and Gap26, induced depression, anhedonia, and anxiety-like behaviour [59]. These three connexin inhibitors, CBX, Gap26, and Gap27, inhibit both astroglial Cx43-containing gap junction and hemichannel [134]. However, lacosamide, which inhibits astroglial hemichannel activity without affecting Cx43 synthesis, led to a mild but antidepressant-like effect rather than depressive mood induction [116,117,118]. Therefore, although astroglial hemichannel inhibition and activation probably contribute to antidepressant-like and antimanic action, respectively, a major mechanism underlying the mood-stabilising effects might be the maintenance of homeostasis through the activation of the gap junction function.

## 8. Conclusions

The pathophysiology of depression associated with inhibition of the astroglial Cx43 hemichannel has been established. Conventional antidepressants, monoaminergic transporter-inhibiting antidepressants and ketamine/esketamine suppress astroglial hemichannel activity. The inhibitory effects on the Cx43 hemichannel contribute to the antidepressive action of these two classes of antidepressants. The question of whether the antidepressant effect is mediated directly by astroglial Cx43 hemichannel suppression or secondary enhancement of Cx43 gap junction induced by (phosphorylated) hemichannel inhibition led to the search for a candidate mechanism responsible for the development of pathophysiology of Cx43-associated antidepressant strategies.

The current understanding of the mechanism of action of mood-stabilising antipsychotics suggests that activation of astroglial Cx43 hemichannel with increased Cx43 synthesis plays an important role in the efficacy of mood-stabilising antipsychotics for the treatment of the acute phase of bipolar disorder. However, the inhibition of Cx43 hemichannel activity with gap junction or Cx43 synthesis probably contributes to the depressive mood induction or antimanic action of some anticonvulsants. These opposite effects of mood-stabilising antipsychotics and depressive mood-inducing anticonvulsants on Cx43 suggest the discrepancy between the pathomechanism and pathophysiology of bipolar disorders. In other words, to develop a robust pathophysiological hypothesis of bipolar disorder associated with astroglial Cx43, several unknown mechanisms remain to be clarified. In order to clarify the more detailed pathomechanisms of bipolar disorder and pathophysiology of mood-stabilising actions, the effects of the mood-stabilising antipsychotics olanzapine, quetiapine, etc., and the mood-stabilising anticonvulsants valproate, carbamazepine, and lacosamide on astroglial Cx43 expression and the function of the Cx43 hemichannel and gap junction should be studied with both in vivo and in vitro preclinical studies.

## Figures and Tables

**Table 1:** Abnormalities of Cx43 expression of patients with mood disorders.

Subject	Region (Cell)	Effect	Reference
Suicide	dorsal lateral prefrontal cortex (astrocyte)	decrease (mRNA)	[50]
Major depression	locus coeruleus	decrease (mRNA)	[49]
Major depression	orbitofrontal cortex	decrease (protein)	[52]
Major depression	prefrontal cortex	decrease (mRNA)	[72]
Major depression (suicide)	Neocortex, mediodorsal thalamus, caudate nucleus, cerebellum	decrease (mRNA)	[51]

We searched MEDLINE using the keywords “(((connexin43) OR (microdissection)) AND ((suicide) OR (depression)) AND (humans) AND ((brain) OR (glia)))” for papers published by 1 November 2020. Relevant articles were obtained in full and assessed for inclusion independently by reviewers. Disagreements among reviewers were resolved via discussion to reach a consensus.

**Table 2:** Abnormalities of Cx43 expression and function of Cx43 in experimental models of depression.

Model	Region (Cell)	Effect	Reference
**(In Vivo)**			
chronic unpredictable stress	prefrontal cortex (rat, in vivo)	decrease (mRNA and protein) suppresses gap junction permeability	[59,60,61]
	Hippocampus (rat, in vivo)	decrease (protein) suppresses gap junction permeability	[62]
acute restraint stress (2 h)	Hippocampus (mouse, in vivo)	No effect (protein) enhances hemichannel permeability	[63]
chronic restraint stress (2 h × 10 times)	Hippocampus (mouse, in vivo)	No effect (protein) enhances hemichannel permeability	[63]
Mouse corticosterone (5 mg/kg/day for 28 days)	Hippocampus (mouse, in vivo)	No effect (protein) increase (phosphorylated protein)	[64]
**(In Vitro)**			
corticosterone (50 µM for 24 h)	cortical astrocyte (rat, in vitro)	decrease (protein in total lysate and plasma membrane) increase phosphorylated Cx43 in plasma membrane supresses gap junction permeability	[68]
Corticosterone (50 µM for 24 h)	hippocampal astrocyte (rat, in vitro)	decrease (protein in total lysate and plasma membrane) increase phosphorylated Cx43 in plasma membrane supresses gap junction permeability	[68]
corticosterone (5‒50 µM for 16 days)	cortical astrocyte (rat, in vitro)	decrease (protein)	[65]
Mouse lipopolysaccharide (1 µg/mL for 24 h)	cortical astrocyte (mouse, in vitro)	augmentation of hemichannel permeability	[75]

We searched MEDLINE using the keywords “(((connexin) OR (hemichannel) OR (gap junction)) AND ((unpredictable stress) OR (restraint stress) OR (corticosterone)))” for papers published by 1 November 2020.

**Table 3:** Summary of the effects of first-line antidepressants, selective serotonin reuptake inhibitors (SSRI), serotonin norepinephrine reuptake inhibitors (SNRI), other monoamine transport inhibitors, norepinephrine reuptake inhibitors (NRI), and nonselective monoamine transporter inhibitors on the expression and function of Cx43.

Agent (Class)	Model (Region)	Treatment (Dose, Duration)	Effect (Function)	Reference
Fluoxetine (SSRI)	Rat (frontal)	in vivo (20 mg/kg for 21 days)	Increase (protein)	[76]
	rat (frontal)	in vivo (10 mg/kg for 21 days)	increase (mRNA/protein) (gap junction: no effect)	[59]
	Rat (frontal) chronic unpredictable stress	in vivo (10 mg/kg for 21 days)	increase (mRNA/protein) (gap junction: augmentation)	[59]
	astrocytoma cells (1321N1/U87MG)	in vitro (30‒60 µM for 24 h)	increase (mRNA/protein)	[80]
	Mouse (cortical astrocyte)	in vitro (10 µM for 24 h)	no effect (protein) (gap junction: inhibition)	[75]
	Mouse lipopolysaccharide (cortical astrocyte)	in vitro (10 µM for 24 h)	(hemichannel: inhibition)	[75]
	mouse exogenous corticosterone (hippocampus)	in vivo (18 mg/kg for 28 days)	Decreased (phosphorylated protein)	[64]
Fluvoxamine (SSRI)	rat (cortical astrocyte)	in vitro (25 µM for 48 h)	increase (protein)	[77]
Paroxetine (SSRI)	Mouse (cortical astrocyte)	in vitro (5 µM for 24 h)	no effect (protein) (gap junction: augmentation)	[75]
	Mouse lipopolysaccharide (cortical astrocyte)	in vitro (5 µM for 24 h)	(hemichannel: inhibition)	[75]
Reboxetine (NRI)	Mouse (cortical astrocyte)	in vitro (10 µM for 24 h)	no effect (protein) (gap junction: no effect)	[75]
	mouse lipopolysaccharide (cortical astrocyte)	in vitro (10 µM for 24 h)	(hemichannel: inhibition)	[75]
Duloxetine (SNRI)	Rat (frontal)	in vivo (10 mg/kg for 21 days)	increase (mRNA/protein) (gap junction: no effect)	[59]
	Rat (frontal) chronic unpredictable stress	in vivo (10 mg/kg for 21 days)	increase (mRNA/protein) (gap junction: augmentation)	[59]
	mouse (cortical astrocyte)	in vitro (5 µM for 24 h)	no effect (protein) (gap junction: no effect)	[75]
	mouse lipopolysaccharide (cortical astrocyte)	in vitro (5 µM for 24 h)	(hemichannel: inhibition)	[75]
Venlafaxine (SNRI)	mouse (cortical astrocyte)	in vitro (5 µM for 24 h)	no effect (protein) (gap junction: inhibition)	[75]
	mouse lipopolysaccharide (cortical astrocyte)	in vitro (5 µM for 24 h)	(hemichannel: inhibition)	[75]
Milnacipran (SNRI)	rat cortical astrocyte	in vitro (25 µM for 48 h)	no effect (protein)	[77]
Cocaine	rat cortical astrocyte	in vitro (100 µM for 48 h)	no effect (protein)	[77]
(nonselective monoamine transporter inhibitor)				

We searched MEDLINE using the keywords “(((connexin) OR (hemichannel) OR (gap junction)) AND ((antidepressant) OR (psychotropic drugs)))” for papers published by 1 November 2020. Relevant articles were obtained in full and assessed for inclusion independently by reviewers. Disagreements among reviewers were resolved via discussion to reach a consensus.

**Table 4:** Summary of the effects of antipsychotics and ketamine on the expression and function of Cx43.

Agent	Model (Region)	Treatment (Dose, Duration)	Cx43 Expression (Function)	Reference
Haloperidol	Rat (frontal)	in vivo (1.5 mg/kg for 21 days)	decrease (protein)	[76]
	rat cortical astrocyte	in vitro (25 µM for 48 h)	no effect (protein)	[77]
Clozapine	Rat (frontal)	in vivo (20 mg/kg for 21 days)	increase (protein)	[76]
	rat cortical astrocyte	in vitro (30 µM for 7 days)	increase (protein) (activation)	[3,8]
Olanzapine	Rat (frontal)	in vivo (2 mg/kg for 21 days)	no effect (protein)	[76]
Ketamine	mouse cortical astrocyte	in vitro (300 µM for 30 min)	Inhibition (gap junction)	[84]
	mouse cortical astrocyte lipopolysaccharide (200 ng/mL)	in vitro (20 µM for 30 min)	Inhibition (hemichannel)	[84]
	mouse cortical astrocyte TNFα + IL1β (20 ng/mL)	in vitro (50 µM for 30 min)	Inhibition (hemichannel)	[84]

The keywords for search the effects of antipsychotics on Cx43 using MEDLINE were “(((connexin) OR (hemichannel) OR (gap junction)) AND ((psychotropic drugs) OR (antipsychotics)))” for antipsychotics papers published by 1 November 2020. The key words for search the effects of ketamine on Cx43 using MEDLINE were “(((connexin) OR (hemichannel) OR (gap junction)) AND (ketamine))” for papers by 1 November 2020. Relevant articles were obtained in full and assessed for inclusion independently by reviewers. Disagreements among reviewers were resolved via discussion to reach a consensus.

## Data Availability

The data presented in this study are available on request from the corresponding author. The data are not able to be publicly available due to equipment dependent data file.

## Abbreviations

CBX	Carbenoxolone
Cx43	Connexin43
IL 1β	Interleukin 1β
NRI	Norepinephrine reuptake inhibitor
PKB	Protein kinase B
SNRI	Serotonin norepinephrine reuptake inhibitor
SSRI	Selective serotonin reuptake inhibitor
TNF α	Tumour necrosis factor α
VDSC	Voltage-dependent sodium channel

## References

[1] Okada M., Fukuyama K., Shiroyama T., Murata M. (2020). A Working Hypothesis Regarding Identical Pathomechanisms between Clinical Efficacy and Adverse Reaction of Clozapine via the Activation of Connexin43. Int. J. Mol. Sci..

[2] Fukuyama K., Ueda Y., Okada M. (2020). Effects of Carbamazepine, Lacosamide and Zonisamide on Gliotransmitter Release Associated with Activated Astroglial Hemichannels. Pharmaceuticals.

[3] Fukuyama K., Okubo R., Murata M., Shiroyama T., Okada M. (2020). Activation of Astroglial Connexin is Involved in Concentration-Dependent Double-Edged Sword Clinical Action of Clozapine. Cells.

[4] Fukuyama K., Fukuzawa M., Ruri O., Okada M. (2020). Upregulated Connexin 43 Induced by Loss-of-Functional S284L-Mutant alpha4 Subunit of Nicotinic ACh Receptor Contributes to Pathomechanisms of Autosomal Dominant Sleep-Related Hypermotor Epilepsy. Pharmaceuticals.

[5] Fukuyama K., Fukuzawa M., Okada M. (2020). Upregulated and hyperactivated thalamic connexin 43 plays important roles in pathomechanisms of cognitive impairment and seizure of autosomal dominant sleep-related hypermotor epilepsy with S284L-mutant α4 subunit of nicotinic ACh receptor. Pharmaceuticals.

[6] Okada M., Fukuyama K., Shiroyama T., Ueda Y. (2019). Carbamazepine Attenuates Astroglial L-Glutamate Release Induced by Pro-Inflammatory Cytokines via Chronically Activation of Adenosine A2A Receptor. Int. J. Mol. Sci..

[7] Okada M., Fukuyama K., Kawano Y., Shiroyama T., Ueda Y. (2019). Memantine protects thalamocortical hyper-glutamatergic transmission induced by NMDA receptor antagonism via activation of system xc^−^. Pharm. Res. Perspect..

[8] Fukuyama K., Kato R., Murata M., Shiroyama T., Okada M. (2019). Clozapine Normalizes a Glutamatergic Transmission Abnormality Induced by an Impaired NMDA Receptor in the Thalamocortical Pathway via the Activation of a Group III Metabotropic Glutamate Receptor. Biomolecules.

[9] Fukuyama K., Okada M. (2018). Effects of levetiracetam on astroglial release of kynurenine-pathway metabolites. Br. J. Pharm..

[10] Fukuyama K., Hasegawa T., Okada M. (2018). Cystine/Glutamate Antiporter and Aripiprazole Compensate NMDA Antagonist-Induced Dysfunction of Thalamocortical L-Glutamatergic Transmission. Int. J. Mol. Sci..

[11] Fukuyama K., Tanahashi S., Hoshikawa M., Shinagawa R., Okada M. (2014). Zonisamide regulates basal ganglia transmission via astroglial kynurenine pathway. Neuropharmacology.

[12] Yamamura S., Hoshikawa M., Dai K., Saito H., Suzuki N., Niwa O., Okada M. (2013). ONO-2506 inhibits spike-wave discharges in a genetic animal model without affecting traditional convulsive tests via gliotransmission regulation. Br. J. Pharm..

[13] Tanahashi S., Yamamura S., Nakagawa M., Motomura E., Okada M. (2012). Clozapine, but not haloperidol, enhances glial D-serine and L-glutamate release in rat frontal cortex and primary cultured astrocytes. Br. J. Pharm..

[14] Quesseveur G., Gardier A.M., Guiard B.P. (2013). The monoaminergic tripartite synapse: A putative target for currently available antidepressant drugs. Current Drug Targets.

[15] Czeh B., Fuchs E., Wiborg O., Simon M. (2016). Animal models of major depression and their clinical implications. Prog. Neuro-Psychopharmacol. Biol. Psychiatry.

[16] Rajkowska G., Miguel-Hidalgo J.J., Wei J., Dilley G., Pittman S.D., Meltzer H.Y., Overholser J.C., Roth B.L., Stockmeier C.A. (1999). Morphometric evidence for neuronal and glial prefrontal cell pathology in major depression. Biol. Psychiatry.

[17] Ongur D., Drevets W.C., Price J.L. (1998). Glial reduction in the subgenual prefrontal cortex in mood disorders. Proc. Natl. Acad. Sci. USA.

[18] Cotter D., Mackay D., Chana G., Beasley C., Landau S., Everall I.P. (2002). Reduced neuronal size and glial cell density in area 9 of the dorsolateral prefrontal cortex in subjects with major depressive disorder. Cereb. Cortex.

[19] Bowley M.P., Drevets W.C., Ongur D., Price J.L. (2002). Low glial numbers in the amygdala in major depressive disorder. Biol. Psychiatry.

[20] Chana G., Landau S., Beasley C., Everall I.P., Cotter D. (2003). Two-dimensional assessment of cytoarchitecture in the anterior cingulate cortex in major depressive disorder, bipolar disorder, and schizophrenia: Evidence for decreased neuronal somal size and increased neuronal density. Biol. Psychiatry.

[21] Maes M., Yirmyia R., Noraberg J., Brene S., Hibbeln J., Perini G., Kubera M., Bob P., Lerer B., Maj M. (2009). The inflammatory & neurodegenerative (I&ND) hypothesis of depression: Leads for future research and new drug developments in depression. Metab. Brain Dis..

[22] Willner P., Scheel-Kruger J., Belzung C. (2013). The neurobiology of depression and antidepressant action. Neurosci. Biobehav. Rev..

[23] Mulders P.C., van Eijndhoven P.F., Schene A.H., Beckmann C.F., Tendolkar I. (2015). Resting-state functional connectivity in major depressive disorder: A review. Neurosci. Biobehav. Rev..

[24] Rajkowska G., Selemon L.D., Goldman-Rakic P.S. (1998). Neuronal and glial somal size in the prefrontal cortex: A postmortem morphometric study of schizophrenia and Huntington disease. Arch. Gen. Psychiatry.

[25] Selemon L.D., Rajkowska G., Goldman-Rakic P.S. (1998). Elevated neuronal density in prefrontal area 46 in brains from schizophrenic patients: Application of a three-dimensional, stereologic counting method. J. Comp. Neurol..

[26] Czeh B., Simon M., Schmelting B., Hiemke C., Fuchs E. (2006). Astroglial plasticity in the hippocampus is affected by chronic psychosocial stress and concomitant fluoxetine treatment. Neuropsychopharmacology.

[27] Banasr M., Chowdhury G.M., Terwilliger R., Newton S.S., Duman R.S., Behar K.L., Sanacora G. (2010). Glial pathology in an animal model of depression: Reversal of stress-induced cellular, metabolic and behavioral deficits by the glutamate-modulating drug riluzole. Mol. Psychiatry.

[28] Araya-Callis C., Hiemke C., Abumaria N., Flugge G. (2012). Chronic psychosocial stress and citalopram modulate the expression of the glial proteins GFAP and NDRG2 in the hippocampus. Psychopharmacology.

[29] Mergenthaler P., Lindauer U., Dienel G.A., Meisel A. (2013). Sugar for the brain: The role of glucose in physiological and pathological brain function. Trends Neurosci..

[30] Garcia-Caceres C., Quarta C., Varela L., Gao Y., Gruber T., Legutko B., Jastroch M., Johansson P., Ninkovic J., Yi C.X. (2016). Astrocytic Insulin Signaling Couples Brain Glucose Uptake with Nutrient Availability. Cell.

[31] Hertz L., Xu J., Song D., Du T., Li B., Yan E., Peng L. (2015). Astrocytic glycogenolysis: Mechanisms and functions. Metab. Brain Dis..

[32] Rajkowska G. (2000). Postmortem studies in mood disorders indicate altered numbers of neurons and glial cells. Biol. Psychiatry.

[33] Rajkowska G., Halaris A., Selemon L.D. (2001). Reductions in neuronal and glial density characterize the dorsolateral prefrontal cortex in bipolar disorder. Biol. Psychiatry.

[34] Uranova N.A., Vostrikov V.M., Orlovskaya D.D., Rachmanova V.I. (2004). Oligodendroglial density in the prefrontal cortex in schizophrenia and mood disorders: A study from the Stanley Neuropathology Consortium. Schizophr. Res..

[35] Brauch R.A., Adnan El-Masri M., Parker J.C., El-Mallakh R.S. (2006). Glial cell number and neuron/glial cell ratios in postmortem brains of bipolar individuals. J. Affect. Disord..

[36] Butt A.M., Kalsi A. (2006). Inwardly rectifying potassium channels (Kir) in central nervous system glia: A special role for Kir4.1 in glial functions. J. Cell Mol. Med..

[37] Kofuji P., Newman E.A. (2004). Potassium buffering in the central nervous system. Neuroscience.

[38] Takeuchi H., Suzumura A. (2014). Gap junctions and hemichannels composed of connexins: Potential therapeutic targets for neurodegenerative diseases. Front. Cell. Neurosci..

[39] Lapato A.S., Tiwari-Woodruff S.K. (2018). Connexins and pannexins: At the junction of neuro-glial homeostasis & disease. J. Neurosci. Res..

[40] Li Q., Li Q.Q., Jia J.N., Liu Z.Q., Zhou H.H., Mao X.Y. (2019). Targeting gap junction in epilepsy: Perspectives and challenges. Biomed. Pharmacother. Biomed. Pharmacother..

[41] Loewenstein W.R. (1981). Junctional intercellular communication: The cell-to-cell membrane channel. Physiol. Rev..

[42] Chever O., Lee C.Y., Rouach N. (2014). Astroglial connexin43 hemichannels tune basal excitatory synaptic transmission. J. Neurosci. Off. J. Soc. Neurosci..

[43] Ribeiro-Rodrigues T.M., Martins-Marques T., Morel S., Kwak B.R., Girao H. (2017). Role of connexin 43 in different forms of intercellular communication—Gap junctions, extracellular vesicles and tunnelling nanotubes. J. Cell Sci..

[44] Naus C.C., Laird D.W. (2010). Implications and challenges of connexin connections to cancer. Nat. Rev. Cancer.

[45] Wang F., Qi X., Zhang J., Huang J.H. (2020). Astrocytic modulation of potassium under seizures. Neural Regen Res..

[46] Fukuyama K., Okada M. (2020). Age-dependent and sleep/seizure-induced pathomechanisms of autosomal dominant sleep-related hypermotor epilepsy. Int. J. Mol. Sci..

[47] Murphy S., Pearce B. (1988). Eicosanoids in the CNS: Sources and effects. Prostaglandins Leukot Essent Fatty Acids.

[48] Portal B., Delcourte S., Rovera R., Lejards C., Bullich S., Malnou C.E., Haddjeri N., Deglon N., Guiard B.P. (2020). Genetic and pharmacological inactivation of astroglial connexin 43 differentially influences the acute response of antidepressant and anxiolytic drugs. Acta Physiol..

[49] Bernard R., Kerman I.A., Thompson R.C., Jones E.G., Bunney W.E., Barchas J.D., Schatzberg A.F., Myers R.M., Akil H., Watson S.J. (2011). Altered expression of glutamate signaling, growth factor, and glia genes in the locus coeruleus of patients with major depression. Mol. Psychiatry.

[50] Ernst C., Nagy C., Kim S., Yang J.P., Deng X., Hellstrom I.C., Choi K.H., Gershenfeld H., Meaney M.J., Turecki G. (2011). Dysfunction of astrocyte connexins 30 and 43 in dorsal lateral prefrontal cortex of suicide completers. Biol. Psychiatry.

[51] Nagy C., Torres-Platas S.G., Mechawar N., Turecki G. (2017). Repression of Astrocytic Connexins in Cortical and Subcortical Brain Regions and Prefrontal Enrichment of H3K9me3 in Depression and Suicide. Int. J. Neuropsychopharmacol..

[52] Miguel-Hidalgo J.J., Wilson B.A., Hussain S., Meshram A., Rajkowska G., Stockmeier C.A. (2014). Reduced connexin 43 immunolabeling in the orbitofrontal cortex in alcohol dependence and depression. J. Psychiatr. Res..

[53] Okada M., Kawano Y., Fukuyama K., Motomura E., Shiroyama T. (2020). Candidate Strategies for Development of a Rapid-Acting Antidepressant Class That Does Not Result in Neuropsychiatric Adverse Effects: Prevention of Ketamine-Induced Neuropsychiatric Adverse Reactions. Int. J. Mol. Sci..

[54] Fukuyama K., Fukuzawa M., Shiroyama T., Okada M. (2020). Pathogenesis and pathophysiology of autosomal dominant sleep-related hypermotor epilepsy with S284L-mutant alpha4 subunit of nicotinic ACh receptor. Br. J. Pharm..

[55] Okada M., Fukuyama K., Shiroyama T., Ueda Y. (2019). Lurasidone inhibits NMDA antagonist-induced functional abnormality of thalamocortical glutamatergic transmission via 5-HT7 receptor blockade. Br. J. Pharm..

[56] Okada M., Fukuyama K., Okubo R., Shiroyama T., Ueda Y. (2019). Lurasidone Sub-Chronically Activates Serotonergic Transmission via Desensitization of 5-HT1A and 5-HT7 Receptors in Dorsal Raphe Nucleus. Pharmaceuticals.

[57] Okada M., Fukuyama K., Kawano Y., Shiroyama T., Suzuki D., Ueda Y. (2019). Effects of acute and sub-chronic administrations of guanfacine on catecholaminergic transmissions in the orbitofrontal cortex. Neuropharmacology.

[58] Okada M., Fukuyama K., Nakano T., Ueda Y. (2019). Pharmacological Discrimination of Effects of MK801 on Thalamocortical, Mesothalamic, and Mesocortical Transmissions. Biomolecules.

[59] Sun J.D., Liu Y., Yuan Y.H., Li J., Chen N.H. (2012). Gap junction dysfunction in the prefrontal cortex induces depressive-like behaviors in rats. Neuropsychopharmacology.

[60] Miguel-Hidalgo J.J., Moulana M., Deloach P.H., Rajkowska G. (2018). Chronic Unpredictable Stress Reduces Immunostaining for Connexins 43 and 30 and Myelin Basic Protein in the Rat Prelimbic and Orbitofrontal Cortices. Chronic Stress.

[61] Jin C., Wang Z.Z., Zhou H., Lou Y.X., Chen J., Zuo W., Tian M.T., Wang Z.Q., Du G.H., Kawahata I. (2017). Ginsenoside Rg1-induced antidepressant effects involve the protection of astrocyte gap junctions within the prefrontal cortex. Prog. Neuro-Psychopharmacol. Biol. Psychiatry.

[62] Lou Y.X., Wang Z.Z., Xia C.Y., Mou Z., Ren Q., Liu D.D., Zhang X., Chen N.H. (2020). The protective effect of ginsenoside Rg1 on depression may benefit from the gap junction function in hippocampal astrocytes. Eur. J. Pharmacol..

[63] Orellana J.A., Moraga-Amaro R., Diaz-Galarce R., Rojas S., Maturana C.J., Stehberg J., Saez J.C. (2015). Restraint stress increases hemichannel activity in hippocampal glial cells and neurons. Front. Cell. Neurosci..

[64] Quesseveur G., Portal B., Basile J.A., Ezan P., Mathou A., Halley H., Leloup C., Fioramonti X., Deglon N., Giaume C. (2015). Attenuated Levels of Hippocampal Connexin 43 and its Phosphorylation Correlate with Antidepressant- and Anxiolytic-Like Activities in Mice. Front. Cell. Neurosci..

[65] Miguel-Hidalgo J.J., Carter K., Deloach P.H., Sanders L., Pang Y. (2019). Glucocorticoid-Induced Reductions of Myelination and Connexin 43 in Mixed Central Nervous System Cell Cultures Are Prevented by Mifepristone. Neuroscience.

[66] Chi Y., Zhang X., Zhang Z., Mitsui T., Kamiyama M., Takeda M., Yao J. (2016). Connexin43 hemichannels contributes to the disassembly of cell junctions through modulation of intracellular oxidative status. Redox Biol..

[67] Hurtubise J.L., Howland J.G. (2017). Effects of stress on behavioral flexibility in rodents. Neuroscience.

[68] Xia C.Y., Wang Z.Z., Zhang Z., Chen J., Wang Y.Y., Lou Y.X., Gao Y., Luo P., Ren Q., Du G.H. (2018). Corticosterone impairs gap junctions in the prefrontal cortical and hippocampal astrocytes via different mechanisms. Neuropharmacology.

[69] Xia C.Y., Chu S.F., Zhang S., Gao Y., Ren Q., Lou Y.X., Luo P., Tian M.T., Wang Z.Q., Du G.H. (2017). Ginsenoside Rg1 alleviates corticosterone-induced dysfunction of gap junctions in astrocytes. J. Ethnopharmacol..

[70] Lampe P.D., TenBroek E.M., Burt J.M., Kurata W.E., Johnson R.G., Lau A.F. (2000). Phosphorylation of connexin43 on serine368 by protein kinase C regulates gap junctional communication. J. Cell Biol..

[71] Cone A.C., Cavin G., Ambrosi C., Hakozaki H., Wu-Zhang A.X., Kunkel M.T., Newton A.C., Sosinsky G.E. (2014). Protein kinase Cdelta-mediated phosphorylation of Connexin43 gap junction channels causes movement within gap junctions followed by vesicle internalization and protein degradation. J. Biol. Chem..

[72] Nagy C., Suderman M., Yang J., Szyf M., Mechawar N., Ernst C., Turecki G. (2015). Astrocytic abnormalities and global DNA methylation patterns in depression and suicide. Mol. Psychiatry.

[73] Schoenfeld T.J., Kloth A.D., Hsueh B., Runkle M.B., Kane G.A., Wang S.S., Gould E. (2014). Gap junctions in the ventral hippocampal-medial prefrontal pathway are involved in anxiety regulation. J. Neurosci. Off. J. Soc. Neurosci..

[74] Eiland L., Romeo R.D. (2013). Stress and the developing adolescent brain. Neuroscience.

[75] Jeanson T., Pondaven A., Ezan P., Mouthon F., Charveriat M., Giaume C. (2015). Antidepressants Impact Connexin 43 Channel Functions in Astrocytes. Front. Cell. Neurosci..

[76] Fatemi S.H., Folsom T.D., Reutiman T.J., Pandian T., Braun N.N., Haug K. (2008). Chronic psychotropic drug treatment causes differential expression of connexin 43 and GFAP in frontal cortex of rats. Schizophr. Res..

[77] Morioka N., Suekama K., Zhang F.F., Kajitani N., Hisaoka-Nakashima K., Takebayashi M., Nakata Y. (2014). Amitriptyline up-regulates connexin43-gap junction in rat cultured cortical astrocytes via activation of the p38 and c-Fos/AP-1 signalling pathway. Br. J. Pharmacol..

[78] Bennett M.V., Contreras J.E., Bukauskas F.F., Saez J.C. (2003). New roles for astrocytes: Gap junction hemichannels have something to communicate. Trends Neurosci..

[79] Retamal M.A., Froger N., Palacios-Prado N., Ezan P., Saez P.J., Saez J.C., Giaume C. (2007). Cx43 hemichannels and gap junction channels in astrocytes are regulated oppositely by proinflammatory cytokines released from activated microglia. J. Neurosci. Off. J. Soc. Neurosci..

[80] Mostafavi H., Khaksarian M., Joghataei M.T., Hassanzadeh G., Soleimani M., Eftekhari S., Soleimani M., Mousavizadeh K., Hadjighassem M.R. (2014). Fluoxetin upregulates connexin 43 expression in astrocyte. Basic Clin. Neurosci..

[81] Singh J.B., Fedgchin M., Daly E.J., De Boer P., Cooper K., Lim P., Pinter C., Murrough J.W., Sanacora G., Shelton R.C. (2016). A Double-Blind, Randomized, Placebo-Controlled, Dose-Frequency Study of Intravenous Ketamine in Patients With Treatment-Resistant Depression. Am. J. Psychiatry.

[82] DiazGranados N., Ibrahim L.A., Brutsche N.E., Ameli R., Henter I.D., Luckenbaugh D.A., Machado-Vieira R., Zarate C.A. (2010). Rapid resolution of suicidal ideation after a single infusion of an N-methyl-D-aspartate antagonist in patients with treatment-resistant major depressive disorder. J. Clin. Psychiatry.

[83] Berman R.M., Cappiello A., Anand A., Oren D.A., Heninger G.R., Charney D.S., Krystal J.H. (2000). Antidepressant effects of ketamine in depressed patients. Biol. Psychiatry.

[84] Liu X., Gangoso E., Yi C., Jeanson T., Kandelman S., Mantz J., Giaume C. (2016). General anesthetics have differential inhibitory effects on gap junction channels and hemichannels in astrocytes and neurons. Glia.

[85] Cohen M.L., Chan S.L., Way W.L., Trevor A.J. (1973). Distribution in the brain and metabolism of ketamine in the rat after intravenous administration. Anesthesiology.

[86] Idvall J., Ahlgren I., Aronsen K.R., Stenberg P. (1979). Ketamine infusions: Pharmacokinetics and clinical effects. Br. J. Anaesth..

[87] Verdolini N., Hidalgo-Mazzei D., Murru A., Pacchiarotti I., Samalin L., Young A.H., Vieta E., Carvalho A.F. (2018). Mixed states in bipolar and major depressive disorders: Systematic review and quality appraisal of guidelines. Acta Psychiatr. Scand..

[88] Delgado A., Velosa J., Zhang J., Dursun S.M., Kapczinski F., de Azevedo Cardoso T. (2020). Clozapine in bipolar disorder: A systematic review and meta-analysis. J. Psychiatr. Res..

[89] Fornaro M., Carvalho A.F., Fusco A., Anastasia A., Solmi M., Berk M., Sim K., Vieta E., de Bartolomeis A. (2020). The concept and management of acute episodes of treatment-resistant bipolar disorder: A systematic review and exploratory meta-analysis of randomized controlled trials. J. Affect. Disord..

[90] Rey Souto D., Pinzon Espinosa J., Vieta E., Benabarre Hernandez A. (2020). Clozapine in patients with schizoaffective disorder: A systematic review. Rev. Psiquiatr. Salud. Ment.

[91] Yatham L.N., Kennedy S.H., Parikh S.V., Schaffer A., Bond D.J., Frey B.N., Sharma V., Goldstein B.I., Rej S., Beaulieu S. (2018). Canadian Network for Mood and Anxiety Treatments (CANMAT) and International Society for Bipolar Disorders (ISBD) 2018 guidelines for the management of patients with bipolar disorder. Bipolar Disord..

[92] Goodwin G.M., Haddad P.M., Ferrier I.N., Aronson J.K., Barnes T., Cipriani A., Coghill D.R., Fazel S., Geddes J.R., Grunze H. (2016). Evidence-based guidelines for treating bipolar disorder: Revised third edition recommendations from the British Association for Psychopharmacology. J. Psychopharmacol..

[93] Escudero M.A.G., Gutierrez-Rojas L., Lahera G. (2020). Second Generation Antipsychotics Monotherapy as Maintenance Treatment for Bipolar Disorder: A Systematic Review of Long-Term Studies. Psychiatr. Q..

[94] Muhiudeen-Russell I.A., Miller-Hance W.C., Silverman N.H. (2001). Unrecognized esophageal perforation in a neonate during transesophageal echocardiography. J. Am. Soc. Echocardiogr..

[95] Kalinin V.V. (2007). Suicidality and antiepileptic drugs: Is there a link?. Drug Saf.

[96] Piedad J., Rickards H., Besag F.M., Cavanna A.E. (2012). Beneficial and adverse psychotropic effects of antiepileptic drugs in patients with epilepsy: A summary of prevalence, underlying mechanisms and data limitations. CNS Drugs.

[97] Murakami T., Okada M., Kawata Y., Zhu G., Kamata A., Kaneko S. (2001). Determination of effects of antiepileptic drugs on SNAREs-mediated hippocampal monoamine release using in vivo microdialysis. Br. J. Pharm..

[98] Okada M., Kaneko S., Hirano T., Ishida M., Kondo T., Otani K., Fukushima Y. (1992). Effects of zonisamide on extracellular levels of monoamine and its metabolite, and on Ca2+ dependent dopamine release. Epilepsy Res..

[99] Okada M., Hirano T., Kawata Y., Murakami T., Wada K., Mizuno K., Kondo T., Kaneko S. (1999). Biphasic effects of zonisamide on serotonergic system in rat hippocampus. Epilepsy Res..

[100] Kawata Y., Okada M., Murakami T., Mizuno K., Wada K., Kondo T., Kaneko S. (1999). Effects of zonisamide on K+ and Ca2+ evoked release of monoamine as well as K+ evoked intracellular Ca2+ mobilization in rat hippocampus. Epilepsy Res..

[101] Kaneko S., Okada M., Hirano T., Kondo T., Otani K., Fukushima Y. (1993). Carbamazepine and zonisamide increase extracellular dopamine and serotonin levels in vivo, and carbamazepine does not antagonize adenosine effect in vitro: Mechanisms of blockade of seizure spread. Jpn. J. Psychiatry Neurol..

[102] Okada M., Hirano T., Mizuno K., Kawata Y., Wada K., Murakami T., Tasaki H., Kaneko S. (1998). Effects of carbamazepine on hippocampal serotonergic system. Epilepsy Res..

[103] Kawata Y., Okada M., Murakami T., Kamata A., Zhu G., Kaneko S. (2001). Pharmacological discrimination between effects of carbamazepine on hippocampal basal, Ca(2+)- and K(+)-evoked serotonin release. Br. J. Pharm..

[104] Yamamura S., Hamaguchi T., Ohoyama K., Sugiura Y., Suzuki D., Kanehara S., Nakagawa M., Motomura E., Matsumoto T., Tanii H. (2009). Topiramate and zonisamide prevent paradoxical intoxication induced by carbamazepine and phenytoin. Epilepsy Res..

[105] Tanahashi S., Yamamura S., Nakagawa M., Motomura E., Okada M. (2012). Effect of lamotrigine and carbamazepine on corticotropin-releasing factor-associated serotonergic transmission in rat dorsal raphe nucleus. Psychopharmacology.

[106] Okada M., Zhu G., Yoshida S., Kanai K., Hirose S., Kaneko S. (2002). Exocytosis mechanism as a new targeting site for mechanisms of action of antiepileptic drugs. Life Sci..

[107] Garbelli R., Frassoni C., Condorelli D.F., Trovato Salinaro A., Musso N., Medici V., Tassi L., Bentivoglio M., Spreafico R. (2011). Expression of connexin 43 in the human epileptic and drug-resistant cerebral cortex. Neurology.

[108] Das A., Wallace G.C.t., Holmes C., McDowell M.L., Smith J.A., Marshall J.D., Bonilha L., Edwards J.C., Glazier S.S., Ray S.K. (2012). Hippocampal tissue of patients with refractory temporal lobe epilepsy is associated with astrocyte activation, inflammation, and altered expression of channels and receptors. Neuroscience.

[109] Hussein A.M., Ghalwash M., Magdy K., Abulseoud O.A. (2016). Beta Lactams Antibiotic Ceftriaxone Modulates Seizures, Oxidative Stress and Connexin 43 Expression in Hippocampus of Pentylenetetrazole Kindled Rats. J. Epilepsy Res..

[110] Dambach H., Hinkerohe D., Prochnow N., Stienen M.N., Moinfar Z., Haase C.G., Hufnagel A., Faustmann P.M. (2014). Glia and epilepsy: Experimental investigation of antiepileptic drugs in an astroglia/microglia co-culture model of inflammation. Epilepsia.

[111] Sills G.J., Rogawski M.A. (2020). Mechanisms of action of currently used antiseizure drugs. Neuropharmacology.

[112] Miyajima T., Kumada T., Saito K., Fujii T. (2013). Autism in siblings with autosomal dominant nocturnal frontal lobe epilepsy. Brain Dev..

[113] Asioli G.M., Rossi S., Bisulli F., Licchetta L., Tinuper P., Provini F. (2020). Therapy in Sleep-Related Hypermotor Epilepsy (SHE). Curr Treat. Options Neurol..

[114] Ito M., Kobayashi K., Fujii T., Okuno T., Hirose S., Iwata H., Mitsudome A., Kaneko S. (2000). Electroclinical picture of autosomal dominant nocturnal frontal lobe epilepsy in a Japanese family. Epilepsia.

[115] Okada M., Zhu G., Yoshida S., Kaneko S. (2010). Validation criteria for genetic animal models of epilepsy. Epilepsy Seizure.

[116] Arif H., Buchsbaum R., Weintraub D., Pierro J., Resor S.R., Hirsch L.J. (2009). Patient-reported cognitive side effects of antiepileptic drugs: Predictors and comparison of all commonly used antiepileptic drugs. Epilepsy Behav. E&B.

[117] Meador K.J., Loring D.W., Boyd A., Echauz J., LaRoche S., Velez-Ruiz N., Korb P., Byrnes W., Dilley D., Borghs S. (2016). Randomized double-blind comparison of cognitive and EEG effects of lacosamide and carbamazepine. Epilepsy Behav. E&B.

[118] Toniolo S., Di Lorenzo F., Bozzali M., Yogarajah M. (2020). The impact of lacosamide on mood disorders in adult patients with epilepsy: A systematic review. Epilepsy Behav. E&B.

[119] Kanba S., Yagi G., Kamijima K., Suzuki T., Tajima O., Otaki J., Arata E., Koshikawa H., Nibuya M., Kinoshita N. (1994). The first open study of zonisamide, a novel anticonvulsant, shows efficacy in mania. Prog. Neuro Psychopharmacol. Biol. Psychiatry.

[120] Oyamada M., Takebe K., Oyamada Y. (2013). Regulation of connexin expression by transcription factors and epigenetic mechanisms. Biochim. Biophys. Acta.

[121] Yoshida S., Yamamura S., Ohoyama K., Nakagawa M., Motomura E., Kaneko S., Okada M. (2010). Effects of valproate on neurotransmission associated with ryanodine receptors. Neurosci. Res..

[122] Fessler E.B., Chibane F.L., Wang Z., Chuang D.M. (2013). Potential roles of HDAC inhibitors in mitigating ischemia-induced brain damage and facilitating endogenous regeneration and recovery. Curr. Pharm. Des..

[123] Hernandez M., Shao Q., Yang X.J., Luh S.P., Kandouz M., Batist G., Laird D.W., Alaoui-Jamali M.A. (2006). A histone deacetylation-dependent mechanism for transcriptional repression of the gap junction gene cx43 in prostate cancer cells. Prostate.

[124] Ogawa T., Hayashi T., Tokunou M., Nakachi K., Trosko J.E., Chang C.C., Yorioka N. (2005). Suberoylanilide hydroxamic acid enhances gap junctional intercellular communication via acetylation of histone containing connexin 43 gene locus. Cancer Res..

[125] Khan Z., Akhtar M., Asklund T., Juliusson B., Almqvist P.M., Ekstrom T.J. (2007). HDAC inhibition amplifies gap junction communication in neural progenitors: Potential for cell-mediated enzyme prodrug therapy. Exp. Cell Res..

[126] Chen T.Y., Kamali M., Chu C.S., Yeh C.B., Huang S.Y., Mao W.C., Lin P.Y., Chen Y.W., Tseng P.T., Hsu C.Y. (2019). Divalproex and its effect on suicide risk in bipolar disorder: A systematic review and meta-analysis of multinational observational studies. J. Affect. Disord..

[127] Taylor D.M., Cornelius V., Smith L., Young A.H. (2014). Comparative efficacy and acceptability of drug treatments for bipolar depression: A multiple-treatments meta-analysis. Acta Psychiatr. Scand..

[128] Lindstrom L., Lindstrom E., Nilsson M., Hoistad M. (2017). Maintenance therapy with second generation antipsychotics for bipolar disorder—A systematic review and meta-analysis. J. Affect. Disord..

[129] Einoch R., Weinreb O., Mandiuk N., Youdim M.B.H., Bilker W., Silver H. (2017). The involvement of BDNF-CREB signaling pathways in the pharmacological mechanism of combined SSRI- antipsychotic treatment in schizophrenia. Eur. Neuropsychopharmacol. J. Eur. Coll. Neuropsychopharmacol..

[130] Aringhieri S., Kolachalam S., Gerace C., Carli M., Verdesca V., Brunacci M.G., Rossi C., Ippolito C., Solini A., Corsini G.U. (2017). Clozapine as the most efficacious antipsychotic for activating ERK 1/2 kinases: Role of 5-HT2A receptor agonism. Eur. Neuropsychopharmacol. J. Eur. Coll. Neuropsychopharmacol..

[131] Jochim J., Rifkin-Zybutz R.P., Geddes J., Cipriani A. (2019). Valproate for acute mania. Cochrane Database Syst Rev..

[132] Bahji A., Ermacora D., Stephenson C., Hawken E.R., Vazquez G. (2020). Comparative efficacy and tolerability of pharmacological treatments for the treatment of acute bipolar depression: A systematic review and network meta-analysis. J. Affect. Disord..

[133] Reus G.Z., Abelaira H.M., Agostinho F.R., Ribeiro K.F., Vitto M.F., Luciano T.F., Souza C.T., Quevedo J. (2012). The administration of olanzapine and fluoxetine has synergistic effects on intracellular survival pathways in the rat brain. J. Psychiatr. Res..

[134] Walrave L., Vinken M., Leybaert L., Smolders I. (2020). Astrocytic Connexin43 Channels as Candidate Targets in Epilepsy Treatment. Biomolecules.

